# Measurement of Shear Strengths of Cu Films Using Precise Chip Forming

**DOI:** 10.3390/ma15030948

**Published:** 2022-01-26

**Authors:** Jeong-Heon Lee, Jae B. Kwak

**Affiliations:** 1 School of Mechanical System and Automotive Engineering, Chosun University, 309 Pilmun-daero, Gwangju 61452, Korea; shilfi21@chosun.kr; 2 Department of Mechanical Engineering, Chosun University, 309 Pilmun-daero, Gwangju 61452, Korea

**Keywords:** mechanical properties, thin films, shear strength, strain, nano cutting, copper, X-ray diffraction

## Abstract

The mechanical properties of thin films are under-researched because of the challenges associated with conventional experimental methods. We demonstrate a technique for determining the intrinsic shear strength and strain of thin films using a nano-cutting technique based on an orthogonal cutting model with precise control of the cutting system. In this study, electroplated Cu films with thicknesses of 1.5 μm and 5 μm and a sputtered Cu film with a thickness of 130 nm were fabricated to evaluate the mechanical strength. Experiments revealed a shear strength of approximately 310 MPa with a shear strain of 1.57 for the electroplated Cu film and a shear strength of 389 MPa with a shear strain of 2.03 for the sputtered Cu film. In addition, X-ray diffraction analysis was performed to correlate the experimental results.

## 1. Introduction

Thin films are becoming increasingly important for various industrial applications, such as microelectronics and electronic devices. Among the most common reasons for the failure of thin films is surface cracking owing to mechanical loading, such as tension, compression, and impact [1,2]. In particular, Jörg et al. reported that the electrical resistance of Mo thin films as traces is dramatically increased at some amount of elongation under tension loading, attributed to surface crack occurrence [2]. The increasing slimness of electronic devices and flexible printed circuit boards (PCBs) requires thinner Cu traces for PCBs with narrow pitch sizes, which results in reliability issues owing to surface cracks and delamination [3,4]. Consequently, we face challenges in understanding the deformation and failure mechanisms of thin film structures involved in mechanical loading. According to the nature of the material strength, shear dislocations result in deformation against the load. In this study, the idea of a metal chip forming process, the cutting or machining process, is used to quantitatively determine the shear strengths and strains of Cu films through extensive plastic deformation.

Generally, mechanical properties are defined as Young’s modulus, yield strength, tensile strength, etc., and are obtained from tensile or compression tests for bulk materials as established by ASTM standards. However, these conventional methods are not applicable to thin film materials with sub-micrometer thickness. Therefore, many studies have attempted to determine the intrinsic strengths of thin films, including nano-indentation and nano-bulge testing. The nano-indentation method is widely used to obtain film properties, including Young’s modulus and the hardness of thin films through indentation tests with a specified tip [5,6,7,8,9,10,11]. However, this method has limitations when applied to very thin films, owing to the substrate effects and the size of the indenter tip. Ritcher et al. [12], Chen et al. [13], Saha and Nix [14], and Pham and Fang [15] investigated not only the elastic inhomogeneity of thin films depending on various substrates but also the considerable effect of pile-up and sink-in attributed to the strain hardening of the material in nanoindentation tests. In turn, the nano-bulge method measures stress–strain curves through thin film deflection against the pressure applied to the specimen by blowing a uniform distribution load on the specimen. This method can determine the stress–strain curve of the material, thereby obtaining Young’s modulus and tensile strength [16,17]. However, sample preparation and handling issues (i.e., clamping) are problematic. In addition, this method may not be appropriate for ceramic films because of the nature of brittle materials. In this study, a technique for evaluating the shear strength of Cu films was researched using a cutting system and an analysis of the fracture mechanism of the electroplated Cu films.

**Figure 1:**
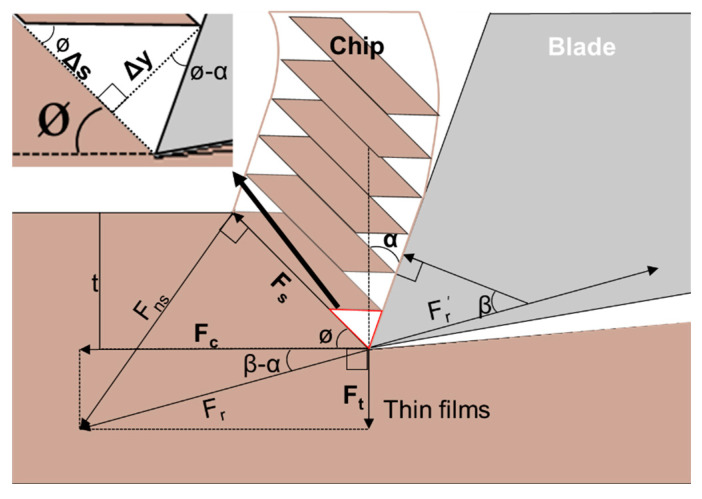
Merchant’s orthogonal cutting model.

Electroplated and sputtered Cu films are considered to be nano-crystalline structures, where surface cracks are typical fracture failures. To determine the shear strength as a fracture resistance of the Cu films, a precise chip-forming process was conducted based on the cutting test suggested by the Merchant’s cutting model [18,19]. According to the Merchant’s cutting model, chip formation is related to the strength of the material when it is subjected to plastic deformation. Consequently, at the root of the chip, a shear angle of 45° develops between the shear plane and the cutting plane, designated as Ø in Figure 1. In this study, films were cut in a diagonal direction from the surface of the film in thickness, as it is practically difficult to cut flat thin films in the horizontal direction. Therefore, the diagonal cutting test was conducted by precisely controlling the blade using two synchronized piezo transducer stages, one in the vertical direction and the other in the horizontal direction. As shown in Figure 1, the blade simultaneously cuts the thin film in both the vertical and horizontal directions, resulting in diagonal cutting. Consequently, orthogonal F_t_ and F_c_ were determined to be resistant forces against diagonal cutting. Figure 1 shows the procedure of the orthogonal cutting, and the chip formation process from cutting with a specific blade geometry, such as the rake angle (α) and width (w). Using the parameters shown in Figure 1, the shear strength ($\tau_{s}$) was determined using Equation (1).

(1) $$\tau_{s}=\frac{F_{s}}{A_{s}}=\frac{\left(F_{c}cos\emptyset -F_{t}sin\emptyset \right)sin\emptyset }{wt},$$

where *F_s_* is the shear force, *A_s_* is the area of the shear plane, *F_c_* is the cutting force in the horizontal direction, *F_t_* is the thrust force in the vertical direction, *Ø* is the angle between the shear plane and the horizontal cutting plane, *w* is the width of the blade, and *t* is the thickness of the cut material.

According to the Merchant’s model, the shear plane angle is associated with both the friction angle and rake angle, which is also related to *F_t_* and *F_c_* and expressed as Equation (2).

(2) $$\emptyset =\frac{\pi }{4}-\frac{1}{2}\left(\beta -\alpha \right)=\frac{\pi }{4}-\frac{1}{2}tan^{-1}\left(\frac{F_{t}}{F_{c}}\right),$$

where *β* is the friction angle between the formed chip and the blade rake surface, and α is the rake angle of the blade.

During the cutting process, the chip is naturally formed as a continuous structure of a group of parallel shear plates. Therefore, the shear strain of the material can be determined from the shear deformation and the thickness of the shear plate, as expressed by Equation (3).

(3) $$\gamma =\frac{\Delta s}{\Delta y}=\frac{cos\alpha }{sin\emptyset cos\left(\emptyset -\alpha \right)},$$

where Δ*s* is the shear deformation in the shear direction, and Δ*y* is the thickness of the primary shear zone, as shown in the upper left corner of Figure 1.

## 2. Experimental

### 2.1. Sample Preparation

In this study, several Cu thin films with different thicknesses were prepared using two fabrication methods: an electroplating process [20] and a magnetron sputtering process [21]. In addition, two types of Si wafer substrates were prepared. One substrate was sputtered by Cu with a thickness of approximately 5 nm for electrical conductivity before the Cu electroplating process, and another substrate was well cleaned with alcohol before magnetron sputtering. For the electroplated Cu film process, CuSO_4_·5H_2_O (0.3 mol/L), H_2_SO_4_ (1.88 mol/L), and HCl (1.7 mmol/L) solvents were mixed as chemical compositions of the electrolyte and diluted with distilled water. Then, 2,3-Dimercapto-1-propanesulfonic acid (2 mg/L) and polyethylene glycol (1 g/L) were added as organic additives. In a bathtub, a stainless-steel cathode and Cu anode were used for electroplating by applying 8 mA/cm^2^ DC at room temperature. Finally, 1.5 μm and 5 μm thickness of electroplated Cu films on substrates were obtained by controlling the deposition time. For the magnetron sputtering process, a polycrystalline Cu material with a two-inch diameter was selected as the sputter target. The vacuum pressure of the deposition chamber was set to 10^−6^ Torr initially, and the deposition proceeded at 4 × 10^−3^ Torr. In the vacuum chamber, high-purity argon gas (purity: 99.999%) was used as an inert gas during the deposition process. The distance between the target and the substrate was approximately 16 cm. The argon plasma applied by DC power at 150 W creates a glow discharge between the target and the substrate at room temperature. Finally, a 130 nm layer Cu film is generated.

### 2.2. Nano Cutting Test

**Figure 2:**
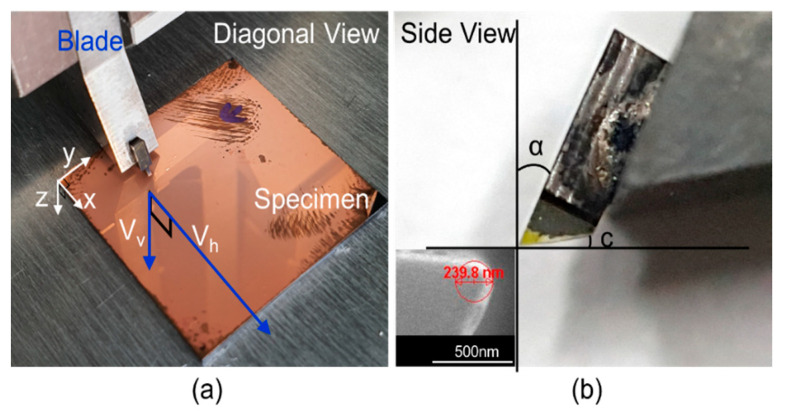
Nano cutting test and blade: (**a**) cutting system with specimen; (**b**) diamond blade and SEM image of the blade edge (lower left).

**Figure 3:**
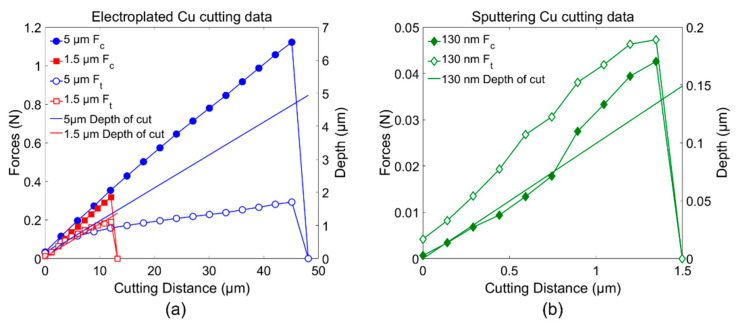
Cutting data for Cu thin films: (**a**) 1.5 μm and 5 μm electroplating cutting; (**b**) 130 nm sputtering cutting.

The strength of the Cu thin films prepared by electroplating and sputtering was evaluated using nano-cutting, as shown in Figure 2a. To cut the Cu film, the blade proceeded diagonally, cutting simultaneously in both the horizontal and vertical directions. For the 5 μm electroplated Cu film, the blade was precisely set to a cutting speed of 500 nm/s in the horizontal (V_h_) and 50 nm/s in the vertical (V_v_) direction. For the 1.5 μm and 130 nm Cu films, the blade was set to a cutting speed of 50 nm/s in the horizontal (V_h_) and 5 nm/s in the vertical (V_v_) direction. The diamond cutting blade has a 2D geometry, with a 20° rake angle (α), 10° clearance angle (c), and a width of 0.3 mm, as shown in Figure 2b. Additionally, the edge radius of the blade measured with a scanning electron microscope (SEM) is approximately 120 nm, which is sharp enough to cut thin films at the submicron scale of thickness [22]. During diagonal cutting, both the cutting force F_c_ and thrust force F_t_ were monitored with individual sensors to determine the shear strengths ($\tau_{s}$). Figure 3a,b show the cutting data for both the 5 μm and 1.5 μm thick electroplated Cu films and the 130 nm thick sputtered Cu film. Three specimens were tested for both the electroplated and sputtered Cu films. According to Figure 3a, although both films have different thicknesses and were cut at different speeds, the graphs show coincidence in cutting behavior, proving the homogeneous mechanical strength attributed to the same electroplating process. Moreover, the depth of the cut graphs shows that 1.5 and 5 μm thicknesses and both *F_t_* and *F_c_* become zero when the blade cuts the Cu film through the thickness to the surface of the wafer substrate. For the 5 μm Cu film, *F_c_* and *F_t_* increased to 1.25 N and 0.25 N, respectively. For the 1.5 μm Cu film, *F_c_* and *F_t_* increased to 0.34 N and 0.2 N, respectively. In addition, both graphs show that *F_c_* linearly increases while *F_t_* tends to saturate at 5.5 μm of a cutting distance, implying that the material cutting behavior changes from elastic to plastic deformation between the blade tip and the Cu film. Therefore, the second slope of the F_t_ data was considered to determine the shear angle and shear strength using Equations (1) and (2), respectively. Considering a very thin film of 130 nm thickness, however, 120 nm of the blade edge radius might be blunt and may cause slipping or plowing before the cutting starts. Figure 3b shows the cutting result for the 130 nm sputtered Cu film. In addition, the depth of the cutting indicates its thickness, and the *F_c_* and *F_t_* values become zero when the blade reaches the surface of the wafer substrate. In this case, both *F_t_* and *F_c_* increase without saturation of the *F_t_* data. This is because the cutting thickness is relatively thin compared to the sharpness of the tool edge and the friction between the blade and the workpiece [20]. The blunt-blade effect may result in a lower shear angle, yet the shear strength is still considerable because the sputtered Cu film is cut.

### 2.3. SEM (Scanning Electron Microscopy) and X-ray Diffraction (XRD) Test

The cracks or pores may be considerable defects of the Cu films and affect the shear strengths if a significant residual stress occurred after the fabrication process. As surface characterization, therefore, SEM (S-4800, Hitachi, Gwangju, Korea, 15.0 kV) was used to visualize the morphologies and cutting behavior of Cu films.

XRD analysis is a successful technique to determine the crystallites [23,24,25,26]. In particular, Munmun Basak et al. used XRD to quantify the size of crystallites using several models, including the Debye Scherrer method, and verified that the determined crystallite size is very similar to the TEM (Transmission electron microscopy) result [23]. Therefore, the crystallinity of the Cu films was analyzed using XRD (PANalytical, X’pert Pro MRD, Gwangju, Korea, Cu-Kα radiation source (λ = 0.15406 nm)). XRD patterns were recorded from 30° to 80° with a scanning step of 1.0 s at 30 mA and 40 kV. The XRD results provide the crystal structure of the Cu films and the coherent domain size, which is considered as the grain size of the material obtained using the Debye Scherrer equation as Equation (4).

(4) $$D=\frac{K\lambda }{bcos\theta }\;\;\;,$$

where *D* is the coherent domain size, *k* is the shape factor, λ is the X-ray wavelength, *b* is the full width at half maximum, and θ is the Bragg angle.

## 3. Results and Discussion

### 3.1. Shear Strengths of Cu Films

The shear strain on the shear surface during the cutting process is associated with a decrease in the shear angle owing to the thickness reduction, as shown in Figure 1. The decrease in the shear angle occurs when the deformation on the shear surface becomes the largest, which is identified by the shear strain determined using Equation (3). Table 1 lists the mechanical properties of both electroplated and sputtered Cu films, including the shear angle (Equation (2)), strain (Equation (3)), and strength (Equation (1)). As aforementioned, both 1.5 and 5 μm thick electroplated Cu films show similar values. Conversely, the 130 nm thick sputtered Cu film shows a smaller shear angle and larger shear strain and strength compared to the electroplated Cu films. This result is attributed to both the effect of a relatively blunt blade and the difference in microstructure and grain size of the workpieces obtained by electroplating and sputtering processes.

**Figure 4:**
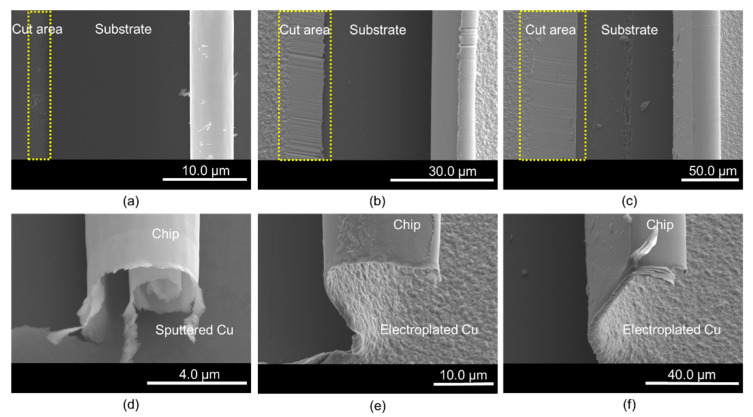
Morphology of cut Cu films: (**a**) top view of the sputtering Cu (130 nm); (**b**) top view of the electroplated Cu (1.5 μm); (**c**) top view of the electroplated Cu (5 μm); (**d**) cutting chip of the sputtered Cu film (130 nm); (**e**) cutting chip of the electroplated Cu film (1.5 μm); (**f**) cutting chip of the electroplated Cu film (5 μm).

Figure 4 shows SEM images of the Cu film specimens after the cutting test. It should be noted that the blade moved from the left to the right in the images. Figure 4a–c identify the cut area, the exposed surface of the substrate, and the rolled chip for the 130 nm sputtered Cu film, and the 1.5 and 5 μm electroplated Cu films, respectively. Figure 4d–f show how the chip is formed and the cutting process. Each length of the cut area corresponds to the cutting distance in Figure 3. The cracks and pores are not observed on the surfaces of the cut area and rolled chips for the three films, which means the residual stresses were low for both film processes.

### 3.2. X-ray Diffraction (XRD) Analysis

**Figure 5:**
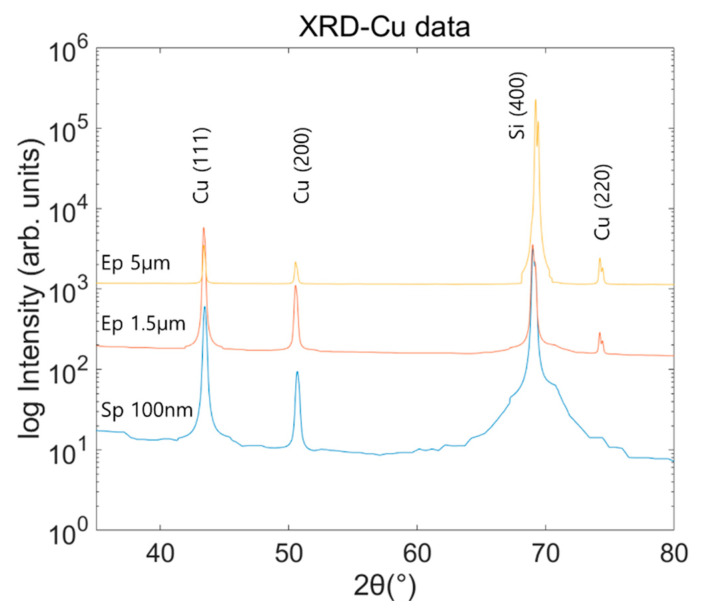
XRD analysis of the tested Cu films.

As shown in Figure 5, the peaks are formed at Bragg angles of 43.4°, 50.5°, 69.2° and 74.2°. It was found that diffraction peaks with strong intensities appear at angles corresponding to (111), (200), and (220) planes of electroplated Cu and (111) and (200) planes for sputtered Cu film. For three films, (400) planes indicate the Si wafer as the substrate. The electroplated Cu films show the same results as conventional Cu films. However, the sputtering Cu thin film has no peak at 74.2°, and the intensity of 50.5° is also weaker than that of electroplated workpieces.

The coherent domain sizes of the Cu thin films were determined through XRD analysis, and the results are listed in Table 2. For both electroplated Cu films, the average domain sizes were similarly determined as 67.0 ± 5.5 nm (5 μm) and 59.1 ± 8.3 nm (1.5 μm), respectively. However, for the sputtered Cu film, the average domain size was determined as 39.3 ± 2.1 nm, which is smaller than that of the electroplated Cu film. According to the Hall-Petch theory, materials with smaller grain sizes have larger strengths [27,28]. This was confirmed by comparing the shear strength of the proposed nano-cutting tests for electroplated and sputtered Cu films. It was found that the shear strength of the sputtered Cu film was approximately 20% higher.

## 4. Conclusions

We report a method to determine the shear strength and strain as mechanical properties of thin films using the nano-cutting technique. In this study, electroplated Cu films with thicknesses of 5 μm and 1.5 μm and sputtered Cu films with a thickness of 130 nm were evaluated. For the electroplated copper thin films of 1.5 and 5 μm, the shear strength was approximately 307 MPa and 313 MPa, and the shear strain was 1.56 and 1.58, respectively. These close results are a result of the same electroplating process. However, for the 130 nm sputtered Cu thin films, the shear strength was found to be 388.8 MPa, and the shear strain was 2.03. This is 20% higher than that of the electroplated Cu thin films. In addition, XRD analysis was conducted to determine the difference in the nanocrystalline structure of the films, and the coherent domain size was obtained using the Debye–Scherrer equation, which is considered as the grain size. The electroplated Cu thin films have a coherent domain size of approximately 60~67 nm, while the sputtering Cu thin films show 39 nm. Therefore, larger shear strength and strain for the sputtered Cu film were expected.

## Figures and Tables

**Table 1:** Cutting results for Cu thin films.

Sample	Test No.	Ø (°)	γ	τ (MPa)
5 μm Electroplated Cu	1	39.3	1.57	302.5
	2	40	1.56	308.4
	3	40.1	1.55	310.3
	Mean Value	39.8 ± 0.4	1.56 ± 0.01	307.1 ± 3.3
1.5 μm Electroplated Cu	1	38.3	1.60	310.3
	2	38.5	1.59	312.5
	3	40.2	1.55	316.1
	Mean Value	39 ± 0.9	1.58 ± 0.02	313.0 ± 2.4
130 nm Sputtered Cu	1	28.3	2.00	359.4
	2	27.3	2.07	416.8
	3	27.8	2.03	390.3
	Mean Value	27.8 ± 0.4	2.03 ± 0.02	388.8 ± 23.5

**Table 2:** Coherent domain size of Cu thin films.

Sample	(hkl)	Coherent Domain Size (nm)
Electroplated Cu 5 μm	(111)	72.4
	(200)	59.5
	(220)	69.2
	Average	67.0 ± 5.5
Electroplated Cu 1.5 μm	(111)	59.4
	(200)	48.8
	(220)	69.2
	Average	59.1 ± 8.3
Sputtered Cu 130 nm	(111)	41.4
	(200)	37.2
	Average	39.3 ± 2.1

## Data Availability

Data sharing is not applicable to this article.
